# Base Excision Repair in Mitotic Cells and the Role of Apurinic/Apyrimidinic Endonuclease 1 (APE1) in Post-Mitotic Transcriptional Reactivation of Genes

**DOI:** 10.3390/ijms252312735

**Published:** 2024-11-27

**Authors:** Suravi Pramanik, Yingling Chen, Kishor K. Bhakat

**Affiliations:** 1 Department of Genetics, Cell Biology and Anatomy, University of Nebraska Medical Center, Omaha, NE 68198, USA; suravi.pramanik@unmc.edu (S.P.); yingling.chen@unmc.edu (Y.C.); 2 Fred & Pamela Buffett Cancer Center, University of Nebraska Medical Center, Omaha, NE 68198, USA

**Keywords:** DNA damage, DNA repair, base excision repair, mitosis, acetylated APE1

## Abstract

Endogenous DNA damage occurs throughout the cell cycle, with cells responding differently at various stages. The base excision repair (BER) pathway predominantly repairs damaged bases in the genome. While extensively studied in interphase cells, it is unknown if BER operates in mitosis and how apurinic/apyrimidinic (AP) sites, intermediates in the BER pathway that inhibit transcriptional elongation, are processed for post-mitotic gene reactivation. In this study, using an alkaline comet assay, we demonstrate that BER is inefficient in mitosis and that AP endonuclease 1 (APE1), a key BER enzyme, is required for the repair of damage post-mitosis. We previously demonstrated that APE1 is acetylated (AcAPE1) in the chromatin. Using high-resolution microscopy, we show that AcAPE1 remains associated with specific regions in the condensed chromatin in each of the phases of mitosis. This association presumably occurs via the binding of APE1 to the G-quadruplex structure, a non-canonical DNA structure predominantly present in the transcribed gene regions. Additionally, using a nascent RNA detection strategy, we demonstrate that the knockdown of APE1 delayed the rapid post-mitotic transcriptional reactivation of genes. Our findings highlight the functional importance of APE1 in the mitotic chromosomes to facilitate faster repair of endogenous damage and rapid post-mitotic gene reactivation in daughter cells.

## 1. Introduction

It is generally accepted that during mitosis, both transcription and DNA repair are temporarily halted [1,2,3]. However, endogenous DNA damage in the genome can occur during mitosis. Apurinic/apyrimidinic (AP) site damage is the most common form of endogenous DNA damage, generated either spontaneously or after the removal of oxidative or alkylated damaged bases by the specific DNA glycosylases [4,5,6,7,8]. It has been shown that the promoter and enhancer regions of most of the actively transcribed genes remain in an open chromatin conformation throughout the cell cycle, even during mitosis, and that these regions are susceptible to endogenous DNA damage, including at AP sites [9,10]. AP sites, if not repaired, inhibit transcription [9,11,12,13]. Thus, to restore the rapid post-mitotic reactivation of genes, repair of AP sites in the transcribed gene regions in mitosis is critical.

AP sites are primarily repaired by AP endonuclease 1 (APE1), which cleaves AP sites and begins their repair by the evolutionarily conserved base excision repair (BER) pathway [5,11,14]. BER is a highly coordinated multi-step cellular process [15]. It is generally initiated by a lesion-specific DNA glycosylase, which cleaves the damaged base, creating an AP site or single-strand break (SSB) [16]. Subsequently, APE1 cleaves the AP site and processes the nicked DNA backbone, thus playing a key role in the BER pathway. The downstream enzyme, DNA polymerase β, fills the nucleotide gap by incorporating a complementary base. Finally, DNA ligase IIIα and XRCC1 together seal the remaining nick in the DNA backbone [17,18,19]. We previously discovered that Lysine 6 and Lysine 7 residues in the N-terminus of APE1 are acetylated (AcAPE1) in chromatin-bound APE1 by histone acetyltransferase p300 [20,21]. APE1 acetylation modulates its AP site cleavage and transcription regulatory activities [21,22]. We have recently shown that AcAPE1 is highly enriched in the transcribed gene regions that contain potential G-quadruplex (G4) structure-forming sequences, and there is a genome-wide association between AcAPE1 and G4 in cells [23]. Additionally, we have shown that APE1 possesses a high binding affinity to G4 structures in vitro [24]. Repair mechanisms of damaged bases or AP sites through APE1-mediated BER are well known in interphase cells. Several earlier studies showed the presence of OGG1, NEIL1, and NEIL2 DNA glycosylases, the initial BER enzymes responsible for removing oxidized bases, in the condensed chromatin [25,26]. However, it is largely unknown whether BER is fully operative in mitosis and how the oxidative/alkylated damaged bases or AP sites that occur in transcribed gene regions are processed during mitosis or in the following cell cycle for the rapid post-mitotic reactivation of genes.

In this study, we demonstrate that AP site damage repair is slow and inefficient in some regions in the genome during mitosis and that APE1 DNA repair activity is important for the post-mitotic repair of damage. AcAPE1 remains associated with the condensed chromatin throughout all the stages of mitosis and overlaps with the transcribed gene regions that contained G4 structures. We further show that nascent RNA production drastically reduces during mitosis, but rapid reactivation of nascent RNA synthesis occurs in post-mitotic cells. We demonstrate that the knockdown of APE1 significantly reduced the activation of nascent RNA production in post-mitotic daughter cells. Our study reveals a novel mechanism for faster repair of endogenous damage in gene regulatory regions for rapid post-mitotic transcription reactivation in daughter cells.

## 2. Results

### 2.1. AcAPE1 Is Associated with the Chromatin Throughout All the Phases of the Cell Cycle

**Figure 1:**
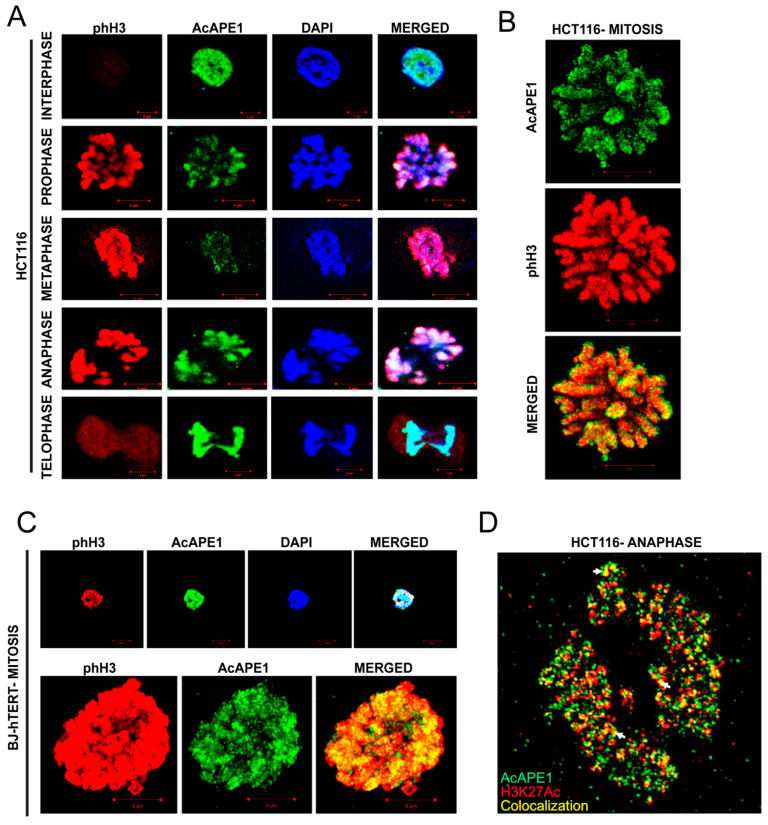
AcAPE1 is associated with the chromatin throughout all the phases of the cell cycle. (**A**) Confocal microscopy images of interphase and mitotic HCT116 colon cancer cells immunostained with α-phH3 and α-AcAPE1 antibodies. Cells were counterstained with DAPI (magnification 63×; scale bars 5 µm). (**B**) Structured illumination microscopy (SIM) images of mitotically arrested HCT116 cell immunostained with α-phH3 and α-AcAPE1 antibodies (magnification 63×; scale bars 5 µm). (**C**) Confocal microscopy (**upper** panel) and SIM (**lower** panel) images of mitotic BJ-hTERT cells immunostained with α-phH3 and α-AcAPE1 antibodies. Cells were counterstained with DAPI (magnification 63×; scale bars 5 µm). (**D**) SIM image of HCT116 cell in anaphase stage of mitosis, immunostained with α-H3K27Ac and α-AcAPE1 antibodies. Yellow color indicates colocalization between H3K27Ac and AcAPE1, as pointed out by white arrows.

We previously generated a highly specific AcAPE1 antibody that recognizes APE1 proteins acetylated at the N-terminal Lysine 6 residue, but not the non-acetylated APE1, both in vitro and in cells [22,27,28,29]. Using this antibody, we demonstrated that, unlike unmodified APE1, AcAPE1 is a chromatin-bound protein in the interphase cells [21]. Immunostaining of HCT116 and BJ-hTERT cells with anti-AcAPE1, anti-phH3 (phospho-histone H3) and anti-tubulin antibodies followed by confocal fluorescence microscopy imaging further shows that AcAPE1 is localized to the condensed chromatin at all the different stages of mitosis (Figure 1A–C, Supplementary Figure S1A–C). High-resolution 3D structured illumination microscopy (SIM) images revealed that AcAPE1 remains localized to specific regions in the condensed chromatin (Figure 1B,C). Studies have shown that even in mitosis, the promoter and enhancer regions of most of the actively transcribed genes maintain specific histone modification marks [9,10]. Consistent with this, we observed AcAPE1 colocalizing with the histone enhancer marker, H3K27Ac, in anaphase HCT116 cells (Figure 1D). We also observed a distinct distribution of unmodified APE1 outside of the chromatin in the mitotic cells (Supplementary Figure S1A,B). Taken together, our observations reveal that AcAPE1 is exclusively associated with the chromatin at all stages of the cell cycle, including the various phases of mitosis, and we speculate that these regions correspond to the transcribed genes.

### 2.2. AcAPE1 Remains Associated with the Promoter/Enhancer Regions During Mitosis Through G4 Structure

**Figure 2:**
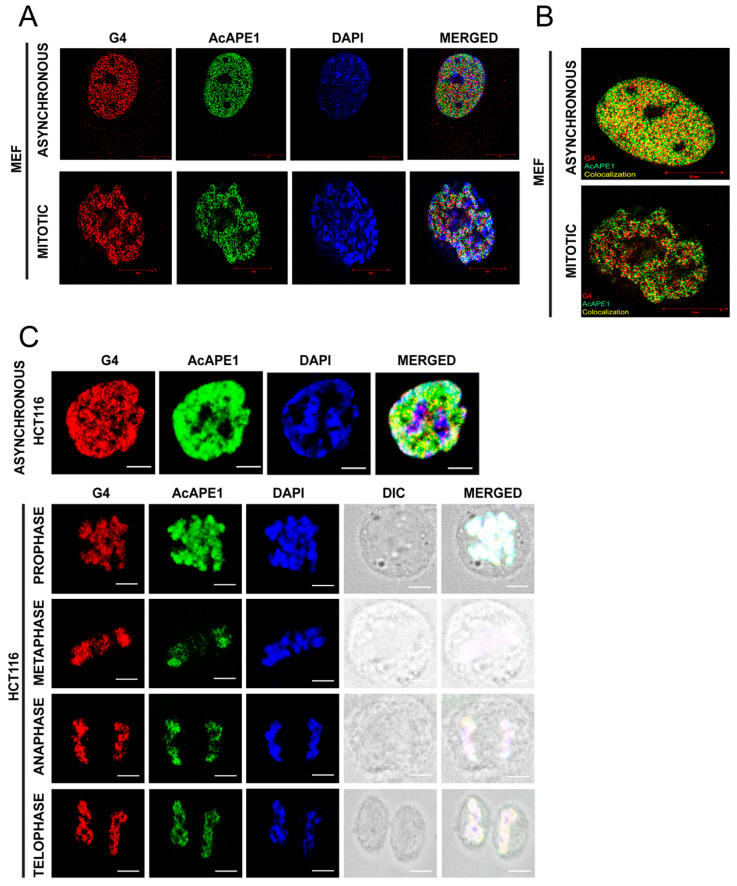
AcAPE1 colocalizes with G4 throughout all the phases of the cell cycle. (**A**) SIM images of asynchronous and mitotic MEF cells immunostained with α-1H6 and α-AcAPE1. Cells were counterstained with DAPI. (**B**) SIM images of MEF cells showing colocalization (yellow) of G4 and AcAPE1 in asynchronous cells and in different stages of mitosis. (**C**) Confocal microscopy images of asynchronous and mitotic (different stages) HCT116 cells immunostained with α-1H6 and α-AcAPE1. Cells were counterstained with DAPI. The scale bar size for Figure 2A is 10 µm (micrometer) and Figure 2C is 5 µm.

We have recently demonstrated that recombinant APE1 can bind to non-canonical DNA secondary structure G4s in vitro with very high affinity, the dissociation constant being in the nanomolar range [24], and AcAPE1 is highly enriched in the transcribed gene regions that contained G4 structures. The distinct binding of AcAPE1 to the condensed chromatin led us to speculate that G4 structures are formed in these regions, which are acting as the docking sites for AcAPE1. We examined the formation of G4 structures during mitosis in multiple cell lines, including mouse embryonic fibroblasts (MEFs) and HCT116 cells, using the G4 structure-specific antibody, clone 1H6. Our confocal microscopy data revealed that, indeed, an appreciable number of G4 structures are formed even in mitosis in the context of condensed chromatin (Figure 2A). We also analyzed the localization of AcAPE1 in these cells using the anti-AcAPE1 immunostaining. Our high-resolution microscopy images and confocal data revealed significant colocalization of AcAPE1 with G4 structures in these cells (Figure 2B,C, Supplementary Figure S2). Together with our previous observation of APE1 possessing high binding affinity to the G4 structure in vitro and our current observation showing the colocalization of AcAPE1 with G4 structures in cells, we conclude that even in mitosis, there exist open chromatin regions where G4 structures can form, and they act as the docking sites for recruiting AcAPE1 to these regions.

### 2.3. BER Is Functional in Mitosis but Damage in Some Regions of the Genome Is Repaired Slowly

**Figure 3:**
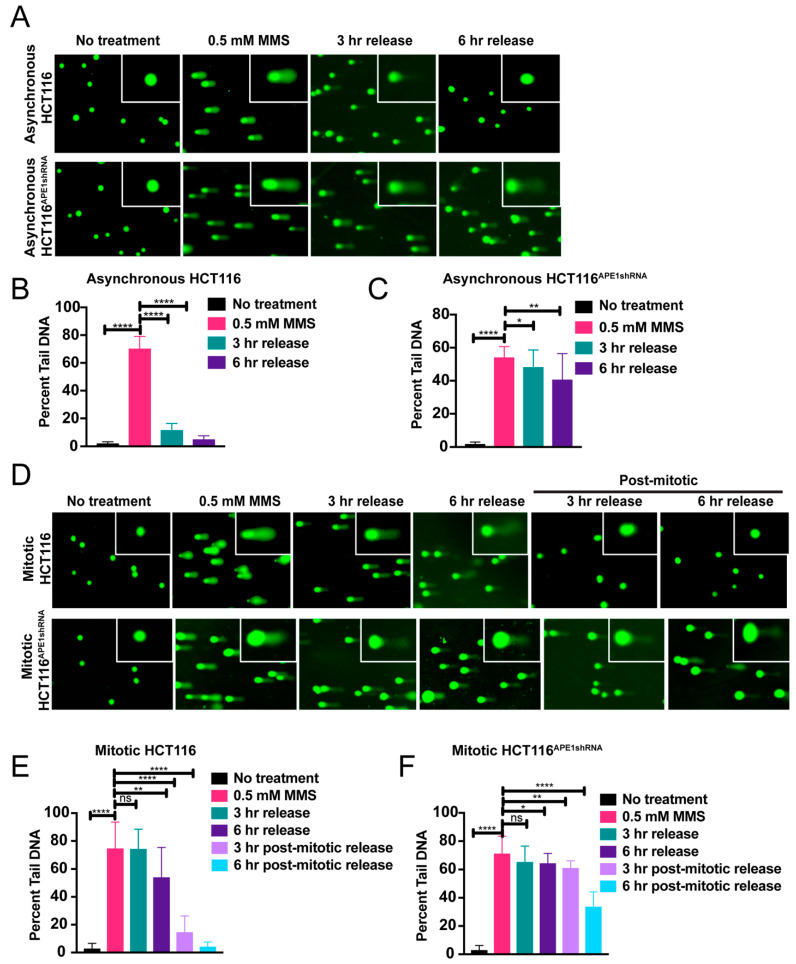
BER is functional but inefficient in mitosis and APE1 is important for the post-mitotic repair. (**A**) Asynchronous HCT116 and APE1-downregulated HCT116^APE1shRNA^ cells were treated with 0.5 mM MMS for 15 min to induce DNA damage and then released to recover for 3 h and 6 h post treatment. Alkaline comet assay was performed with these cells. (**B**) Quantitation of percent tail DNA for asynchronous HCT116 cells (*n* = 100 cells) before and after treatment with MMS. (**C**) Quantitation of percent tail DNA for asynchronous APE1-downregulated HCT116^APE1shRNA^ cells (*n* = 100 cells). (**D**) Mitotically arrested HCT116 and HCT116^APE1shRNA^ cells were treated with 0.5 mM MMS for 15 min and then allowed to recover for 3 h and 6 h post treatment. Alkaline comet assay was performed with or without releasing these cells from mitotic arrest. (**E**) Quantitation of percent tail DNA for mitotic and post-mitotic HCT116 cells (*n* = 100 cells). (**F**) Quantitation of percent tail DNA for mitotic and post-mitotic HCT116^APE1shRNA^ cells (*n* = 100 cells). The *p*-values were determined using an unpaired Student’s *t*-test (**** *p* < 0.0001, ** *p* < 0.01, * *p* < 0.05, ns (nonsignificant) = *p* > 0.05). Error bars denote ± SD.

**Figure 4:**
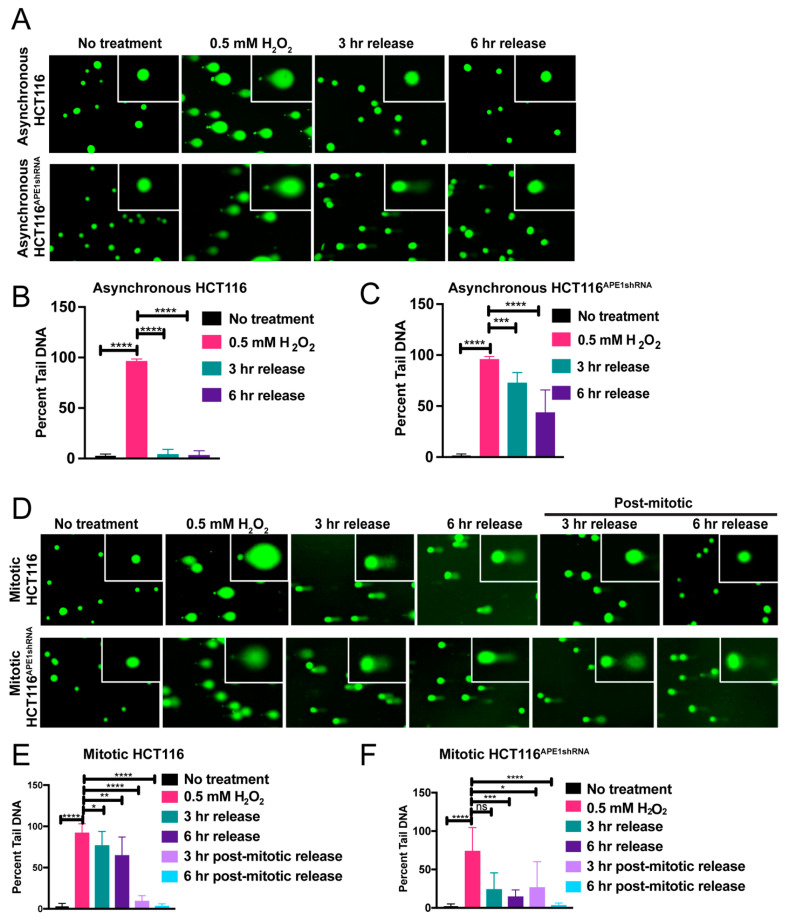
Oxidative damage repair is inefficient in mitosis (**A**) Asynchronous HCT116 and APE1-downregulated HCT116^APE1shRNA^ cells were treated with 0.5 mM H_2_O_2_ for 15 min and released for 3 h and 6 h post treatment. Alkaline comet assay was performed with these cells. (**B**) Quantitation of percent tail DNA for asynchronous HCT116 cells (*n* = 100 cells). (**C**) Quantitation of percent tail DNA for asynchronous APE1-downregulated HCT116^APE1shRNA^ cells (*n* = 100 cells). (**D**) Mitotic arrested HCT116 and HCT116^APE1shRNA^ cells were treated with 0.5 mM H_2_O_2_ for 15 min and then allowed to recover for 3 h and 6 h post treatment. Alkaline comet assay was performed with or without releasing these cells from mitotic arrest. (**E**) Quantitation of percent tail DNA for mitotic and post-mitotic HCT116 cells (*n* = 100 cells). (**F**) Quantitation of percent tail DNA for mitotic and post-mitotic HCT116^APE1shRNA^ cells (*n* = 100 cells). The *p*-values were determined using an unpaired Student’s *t*-test (**** *p* < 0.0001, *** *p* < 0.001, ** *p* < 0.01, * *p* < 0.05, ns (nonsignificant) = *p* > 0.05). Error bars denote ± SD.

While repair of endogenous damage by BER in interphase cells has been very well established, whether BER is operative in mitosis has not been explored. Therefore, we first examined if base damages or AP sites are repaired by BER during mitosis. We used asynchronous and mitotically arrested HCT116 cells (Supplementary Figure S3A) and treated them with DNA damaging agents, including methyl methanesulfonate (MMS) or hydrogen peroxide (H_2_O_2_), which generate damages that are suitable for repair primarily through the BER pathway (Figure 3 and Figure 4). We also performed immunostaining using anti-γH2AX (gamma H2AX), a double-strand break marker, to confirm that MMS treatment to these cells does not generate double-strand breaks (DSBs) (Supplementary Figure S3B). We then released the damaging agent-treated cells in fresh medium for 3 h and 6 h to allow repair and monitored the repair kinetics over the elapsed time frame using the alkaline comet assay. The results clearly indicate that interphase asynchronized cells were able to repair DNA damage induced by MMS or H_2_O_2_ treatment within the 6 hours’ time, as indicated by the marked decrease in the percent tail DNA following 3 h and 6 h of release after damage (Figure 3A (upper panel),B; Figure 4A (upper panel),B). On the contrary, although the mitotic cells were able to repair most of this damage, a significant amount of damage remained as along as cells were kept arrested in mitosis (Figure 3D (upper panel),E; Figure 4D (upper panel),E). However, complete repair of the damage was observed when the cells were released from the mitotic arrest (Figure 3D (upper panel),E; Figure 4D (upper panel),E). These data suggest that during mitosis, BER is functional, but damage repair in some regions in the genome is slow or inefficient compared to other regions.

To further investigate the importance of APE1 in the repair of damage during mitosis, we performed an alkaline comet assay under the same conditions as mentioned above, using APE1-downregulated HCT116^APE1shRNA^ cells. Our results reveal that the knockdown of APE1 significantly reduced the repair of the damage in both the asynchronous and mitotic cells, as indicated by the substantial amount of comet tail DNA still present in these cells even after 3 h and 6 h of releasing them into the fresh medium (Figure 3A,D (lower panel),C; Figure 4A,D (lower panel),F). Taken together, our results indicate that although BER is functional in mitosis, damage in certain regions of the genome is slowly repaired and that APE1 is essential for this repair process.

### 2.4. Loss of APE1 in Cells Affects Nascent RNA Production and Delays Rapid Post-Mitotic Gene Reactivation in Daughter Cells

**Figure 5:**
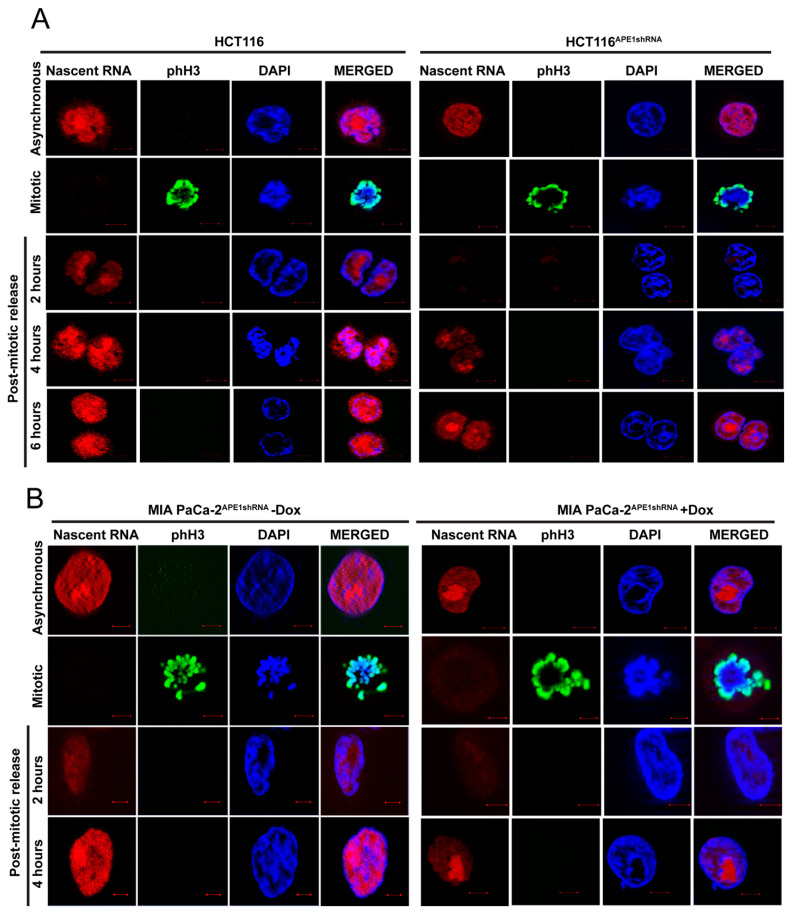
APE1 knockdown reduces nascent RNA production and delays transcriptional reactivation in post-mitotic daughter cells. (**A**) Confocal microscopy images of HCT116 and HCT116^APE1shRNA^ cells immunostained with α-phH3 antibody and nascent RNA labeled with EU under asynchronous, mitotic arrest, and post-mitotic release conditions at different time points. (**B**) Confocal microscopy images of MIA PaCa-2^APE1shRNA^ cells treated without or with Dox, immunostained with α-phH3 antibody, and nascent RNA labeled with EU under asynchronous, mitotic, and different time points of post-mitotic release conditions. Scar bar: 2 µm.

We studied the global impact of the loss of APE1 on transcription by examining the nascent RNA production in APE1 knockdown cells. Nascent RNA production was globally reduced upon the knockdown of APE1. We tested whether, during mitosis, AcAPE1 binding to the regulatory regions of active genes may facilitate repair and the loading of RNA Polymerase II (RNA Pol II) and of the transcription factors for rapid post-mitotic gene reactivation. We examined nascent RNA production in wild-type (WT) and APE1 knockdown HCT116 cells by 5-ethynyl uridine (EU) labeling in mitotically arrested cells, and after release from mitosis. The mitotically arrested cells made little or no nascent RNA. As cells exited mitosis, a time-dependent nascent RNA accumulation was observed, and this was much delayed in the APE1 knockdown cells (Figure 5A,B). Our results indicate that APE1 is important for rapid transcriptional reactivation of genes once the cells exit mitosis.

## 3. Discussion

A vast majority of studies have focused on understanding the cellular DNA damage response (DDR) in interphase cells. However, cells respond to DNA damage differently at different stages of the cell cycle. The DDR signaling and the subsequent damage repair pathways operate differently in interphase and mitotic cells. Understanding the mechanism of damage repair in mitosis is of prime importance since mutations accumulated during mitosis can lead to chromosomal aberrations, genomic instability in daughter cells, senescence, or cell death.

During mitosis, chromatin is condensed, and transcription is thought to be temporarily halted with the dissociation of transcription factors. It has been shown that the promoter and enhancer regions of most of the actively transcribed genes, including *HSP70* and *MYC,* remain in an open chromatin conformation throughout the cell cycle, even during mitosis [30]. These regions are susceptible to endogenous DNA damage by multiple sources, including ROS. Studies have shown the presence of the initial BER proteins, such as many DNA glycosylases, which are involved in the removal of oxidative damaged bases on the mitotic chromosomes. Human OGG1, the major DNA glycosylase responsible for the removal of 8-oxoG, the most common endogenous oxidative base lesion in the DNA, was found to be associated with condensed chromatin during mitosis [25]. Hildrestrand et al. have shown that human NEIL1 DNA glycosylase is associated with condensed mitotic chromatin [26]. However, it was largely unknown whether cells can efficiently remove damaged bases and AP sites that occur in the genome during mitosis and whether BER is fully operative in mitosis. In this study, using an alkaline comet assay, we have provided evidence, for the first time, that BER is functional during mitosis, but repair of damaged bases/AP sites in some regions in mitotic chromosomes is slow and inefficient. We also show that APE1, the second enzyme in the BER pathway, is essential for damage repair during mitosis and facilitates post-mitotic gene reactivation in daughter cells.

Interestingly, our high-resolution microscopic images revealed that the post-translationally modified APE1 (AcAPE1) is constitutively associated with the mitotic chromatin throughout all the phases of mitosis, and these regions colocalize with active enhancer marker H3K27Ac and overlap with G4 structures, which are predominantly present in the transcribed gene regions [31]. Genome-wide mapping using the G4 structure-specific antibody has revealed that endogenous G4s are enriched in the nucleosome-depleted regions (NDRs) upstream of the transcription start sites (TSSs) [32]. Although initially it was thought that G4 formation at promoters is dependent on active transcription-induced torsional stress and negative super helicity of the DNA, a recent study by Shen et al. has demonstrated that chromatin status and not transcription is the sole determinant of promoter G4 folding in the cells [33]. Therefore, it is reasonable to assume that although in mitosis, there is a temporary suspension of transcription, G4 structures can still be formed in the gene regulatory elements that remain in the open chromatin state in this phase of the cell cycle. Consistent with that, in our present study, we could detect G4 structures in the condensed mitotic chromatin using the well-established G4 structure-specific antibody. The persistent presence of AcAPE1 in the gene regulatory regions that overlap with G4 raises the possibility that either these regions are more prone to DNA damage or APE1 resides in the transcribed gene region by binding to G4 structures throughout the cell cycle, including in mitosis. This enhances the local concentration and the availability of APE1 near the damaged sites to promote faster repair to facilitate gene expression in post-mitotic daughter cells. Several lines of evidence support the latter hypothesis. First, APE1 can bind with high affinity to G4 structures without damage in vitro. Second, genome-wide mapping revealed that AcAPE1 is highly enriched in the promoter/enhancer transcriptionally active regions and overlaps with DNA sequences that form G4 structures in interphase cells as well in mitotic cells. Third, the knockdown of APE1 significantly affected post-mitotic gene reactivation, as observed by delayed nascent RNA production.

Previous studies examining DDR activation and repair in mitosis have mostly focused on DNA DSBs, which may be primarily repaired by homologous recombination (HR) and non-homologous end joining (NHEJ) in interphase cells [34]. It was shown that the induction of DNA DSBs in mitotic chromosomes induces rapid and efficient recruitment of the early protein Mre11-Rad50-Nbs1 (MRN) complex, which recognizes the DSBs by tethering the broken ends, but subsequent recruitment of downstream protein does not occur as long as cells are in mitosis [35]. Recruitment of the subsequent downstream enzymes of the NHEJ pathway does not happen until the daughter cells enter the subsequent G1 phase when the repair is completed [36,37]. Thus, it was suggested that repair of DSBs can be initiated but cannot be completed in mitotic cells [36,38]. Interestingly, forceful activation of the complete repair of DSBs during mitosis led to the mis-segregation of chromosomes and telomere fusion [39,40]. This inhibition of HR and NHEJ during mitosis plays a genome-protective role. Therefore, cells have evolved mechanisms to robustly silence downstream DDR processes during mitosis to prevent aberrant NHEJ of the broken DNA ends and unprotected telomeres, which would lead to mitotic catastrophe.

Unlike the repair of DSBs, our study demonstrates that mitotic cells were able to repair the majority of AP sites/SSBs, but a significant amount of damage remained if cells were kept arrested in mitosis, suggesting that repair in some regions in the genome is either slow or refractory during mitosis. We speculate that these regions correspond to condensed heterochromatin regions. A possible explanation for the inefficient BER in these regions in mitosis could be the lack of accessibility or recruitment of the upstream DNA glycosylase or proteins downstream of APE1 in the condensed chromatin to complete the process efficiently. More studies are warranted to investigate the damage repair kinetics in the non-transcribed heterochromatin versus euchromatin regions in both interphase and mitotically arrested cells.

The process of transcriptional reactivation plays a crucial role in establishing the identity of daughter cells after mitosis [41]. For efficient transcription to occur, the loading of the transcriptional machinery, including RNA Pol II, to the gene regulatory regions is essential. As AP sites or SSBs block elongation by RNA Pol II and inhibit transcription, faster repair of these damages in the transcribed regions both in interphase cells and in mitosis is critical for post-mitotic reactivation of gene transcription or expression. Therefore, we speculate that AcAPE1 remains associated with transcribed gene regions in the mitotic chromatin through G4 binding, and its preloading to these regions facilitates faster damage repair and post-mitotic transcription reactivation. Supporting this, we have shown that AcAPE1 colocalizes with enhancer markers H3K27Ac and G4s, both of which are predominantly present in the transcribed gene regions. Notably, we have provided direct evidence that downregulation of APE1 significantly affected post-mitotic gene reactivation, as observed by the delay in nascent RNA production in APE1 knockdown cells.

Overall, our study suggests a novel mechanism in which AcAPE1 is consistently recruited to the gene regulatory elements in mitosis through G4 structures that enable the repair of DNA damages in these open chromatin regions. This repair is important for the recruitment of the transcriptional machinery, which eventually facilitates post-mitotic transcriptional reactivation of the genes that are essential for the daughter cells’ survival.

## 4. Materials and Methods

### 4.1. Reagents

Primary antibodies used for the immunofluorescence studies were mouse monoclonal anti-APE1 (1:100; Novus Biologicals, Littleton, CO, USA, Cat # NB100-116), mouse phospho-histone H3 (Ser10) (1:100; Cell Signaling Technology, Danvers, MA, USA, Cat # 9706), anti-AcAPE1 (1:50) and mouse monoclonal anti-G4-clone 1H6 (1:50; Millipore Sigma, Burlington, MA, USA, Cat # MABE1126). Secondary antibodies used for the immunofluorescence studies were Alexa Fluor 594 goat anti-mouse IgG (H+L) (1:500; Life Technologies Corporation, Carlsbad, CA, USA, Cat # A11005) or Alexa Fluor 488 goat anti-rabbit IgG(H+L) (1:500; Life Technologies Corporation, Cat # A11008). Reagents used in the study include RNase A from bovine pancreas (Sigma, Kawasaki, Japan, Cat # R4875-100MG), methyl methanesulfonate (Sigma-Aldrich, St. Louis, MO, USA, Cat # M4016), hydrogen peroxide solution (Sigma-Aldrich Cat # H1009), thymidine (Sigma-Aldrich Cat # 855006), and nocodazole (Sigma-Aldrich Cat # M1404). For the alkaline comet assay, CometAssay^TM^ Kit (Trevigen Inc., Gaithersburg, MD, USA; Cat # 4250-050-K) and SYBR^TM^ Gold (ThermoFisher Scientific, Waltham, MA, USA; Cat # S11494) were used. For nascent RNA detection in the cells, a Click-iT^TM^ RNA Alexa Fluor 594 Imaging kit (ThermoFisher Scientific; Cat # C10330) was used.

### 4.2. Biological Resources

The human mutant KRAS expressing pancreatic ductal adenocarcinoma MIA PaCa-2 (ATCC, Manassas, VA, USA, Cat # CRM-CRL-1420) cell line, mouse embryonic fibroblasts (MEF) cells, and BJ-5ta (BJ-hTERT; ATCC, Manassas, VA, USA, Cat # CRL-4001) cells were all maintained in high-glucose Dulbecco’s Modified Eagle’s Medium (DMEM; ThermoFisher Scientific, Cat # 11965084) supplemented with 10% fetal bovine serum (FBS; Sigma, Catalog # F2442) and an antibiotic mixture of 100 U/mL penicillin and 100 µg/mL streptomycin (Gibco-BRL, Grand Island, NY, USA). The human colon cancer cell line HCT116 (ATCC, Cat # CCL-247) was maintained in McCoy’s 5A (modified) medium (Gibco; Grand Island, NY, USA, Cat # 16600082) supplemented with 10% fetal bovine serum and an antibiotic mixture of 100 U/mL penicillin and 100 µg/mL streptomycin. For APE1 knockdown studies, HCT116 cells showing stable expression of APE1-shRNA (HCT116^APE1shRNA^) (kind gift from Dr. Sheila Crowe, University of California, San Diego, CA, USA) were cultured in a medium similar to that used for the HCT116 cells with the addition of Puromycin Dihydrochloride (Gibco, Cat # 12122530) at a final concentration of 1 µg/mL. The APE1 knockdown MIA PaCa-2 cell line was generated as described in a previous publication [24] All cell lines were authenticated by STR DNA profiling by Genetica DNA Laboratories, Burlington, NC, USA.

### 4.3. Immunofluorescence

Cells were cultured on coverslips (Fisherbrand, Pittsburg, PA, USA, Cat # 12-542-B) and fixed in 4% paraformaldehyde (PFA; Sigma) in PBS for 15 min at room temperature (RT). Cells were subsequently permeabilized and blocked for 1 h at RT using a blocking buffer containing 0.5% Triton X-100 (Sigma), 10% goat serum (Thermo-Fisher, Waltham, MA, USA # 50062Z), glycine (Fisher BioReagents, Pittsburg, PA, USA, Cat # BP381-1), and sodium azide in PBS. After permeabilization and blocking, cells were incubated in a blocking buffer with primary antibodies overnight at 4 °C and, subsequently, the corresponding secondary antibodies for 1 h at RT. Cells were then washed in PBS and mounted using mounting media with DAPI (Vector Laboratories- Newark, CA, USA, Item # VV-93952-27). For G-quadruplex (G4) staining, an additional permeabilization step was performed after PFA fixation by incubating cells in PBS containing 0.5% Tween 20 (Fisher BioReagents, Cat # BP337-100) for 20 min at 37 °C. Following Tween 20 permeabilization, cells were treated with 0.04 µg/µL RNase A and subsequently blocked and processed as described above. For the DNase experiments, cells were fixed in 4% PFA in PBS for 30 min, permeabilized with 0.2% Triton X-100 in PBS for 1 min, and washed in PBS at RT. Cells were then incubated for 2 h at 37 °C in DNase reaction buffer with or without 0.06 U/µL of DNase I (RQ1 DNase, Promega, Madison, WI, USA). The working DNase reaction buffer contained 40 mM Tris-HCl (pH 8), 5 mM CaCl_2_, 2 mM MgCl_2_, and 100 µg/mL BSA. Following DNase digestion, the cells were washed in PBS and stained as described above. All immunofluorescence images were captured by confocal microscopy or super-resolution (~100 nm) structured illumination microscopy (SIM), where indicated. Confocal microscope images were captured using the Zeiss LSM 800 with Airyscan Microscope, While Plains, NY, USA in the Advanced Microscopy Core Facility (AMCF) at the University of Nebraska Medical Center (UNMC), Omaha, NE, USA. Three-dimensional (3D) SIM images were collected with the Zeiss ELYRA PS.1 Super-Resolution Microscope, While Plains, NY, USA in the AMCF at UNMC.

### 4.4. Cell Synchronization in Mitosis

Double thymidine followed by nocodazole treatment was used to arrest the cells in mitosis. Cells were cultured in respective media till the plates were about 40% confluent. Thymidine stock solution was prepared in sterile water (200 mM) and was added to the medium at a final concentration of 2 mM. Cells were incubated at 37 °C for 16 h. Thymidine-containing medium was discarded, plates were rinsed once with sterile PBS, and fresh medium was added to the cells for 9 h at 37 °C. Thymidine was again added to the medium (final concentration of 2 mM) and the cells were incubated at 37 °C for another 16 h. The thymidine-containing media were then discarded, and the plates were rinsed once with sterile PBS. Fresh medium containing 100 ng/mL nocodazole (stock concentration: 2 mg/mL in DMSO) was added to the cells for 12 h and incubated at 37 °C to arrest the cells at mitosis. These cells were used for further experiments alongside an asynchronous culture of the same cell line. Each time, cell synchronization in mitosis was confirmed by flow cytometry by measuring the percentage of cells in the G2/M phase of the cell cycle.

### 4.5. Alkaline Comet Assay

Asynchronous, mitotic, and post-mitotic (released from double thymidine and nocodazole block) cells were cultured in 6-well plates followed by treatment with either 0.5 mM methyl methanesulfonate (MMS) for 15 min or 0.5 mM hydrogen peroxide (H_2_O_2_) for 15 min. The no treatment and treated groups were trypsinized and collected in PBB immediately following 15 min treatment and pelleted and processed for alkaline comet assay. For the 3 h and 6 h release groups, the DNA-damaging-agent-containing media were discarded, the cells were washed once in sterile PBS, and fresh medium was added. These cells were then left in the 37 °C incubator to recover from the damage for 3 h and 6 h prior to collecting and processing them for comet assay. For the alkaline comet assay, a CometAssay^TM^ Kit by Trevigen Inc. was used, and the assay was performed according to the manufacturer’s protocol. The DNA from the cells after electrophoresis was stained with SYBR Gold Nucleic Acid Gel Stain (10,000X Concentrate in DMSO), and the images were captured using the Zeiss LSM 800, While Plains, NY, USA with Airyscan Microscope in the Advanced Microscopy Core Facility (AMCF) at the University of Nebraska Medical Center (UNMC). Image analysis and quantification of the percent tail DNA and tail moment were performed in ImageJ software v1.3.1 using the OpenComet, Austin, TX, USA (http://www.cometbio.org/index.html, accessed on 1 January 2024) plug-in. At least 100 cells or comets were analyzed per sample for quantification, statistical analysis was performed, and the data were plotted in GraphPad Prism 8 software.

### 4.6. Nascent RNA Detection in Cells

For nascent RNA detection in the cells, a Click-iT RNA Alexa Fluor 594 Imaging Kit was used. Asynchronous, mitotic, and post-mitotic cells were cultured on coverslips and treated for 1 h with 1 mM of 5-ethynyl uridine (EU) and were subsequently processed for the nascent RNA labeling according to the manufacturer’s protocol. After nascent RNA labeling, the cells were further processed for the immunofluorescence experiment to detect phospho-histone H3 (Ser10). The images were acquired using the Zeiss LSM 800, While Plains, NY, USA with Airyscan Microscope.

## Data Availability

All the data are contained within the manuscript.

## Supplementary Materials

The following supporting information can be downloaded at https://www.mdpi.com/article/10.3390/ijms252312735/s1.
