# Supplementary material for: Base Excision Repair in Mitotic Cells and the Role of Apurinic/Apyrimidinic Endonuclease 1 (APE1) in Post-Mitotic Transcriptional Reactivation of Genes
Source: Int J Mol Sci. 2024 Nov 27;25(23):12735. doi: 10.3390/ijms252312735 (PMC11641725; doi:10.3390/ijms252312735)
Supplement: Supplementary file 1 [file ijms-25-12735-s001.zip › ijms-3325146-supplementary.pdf]

Supplementary Figures:

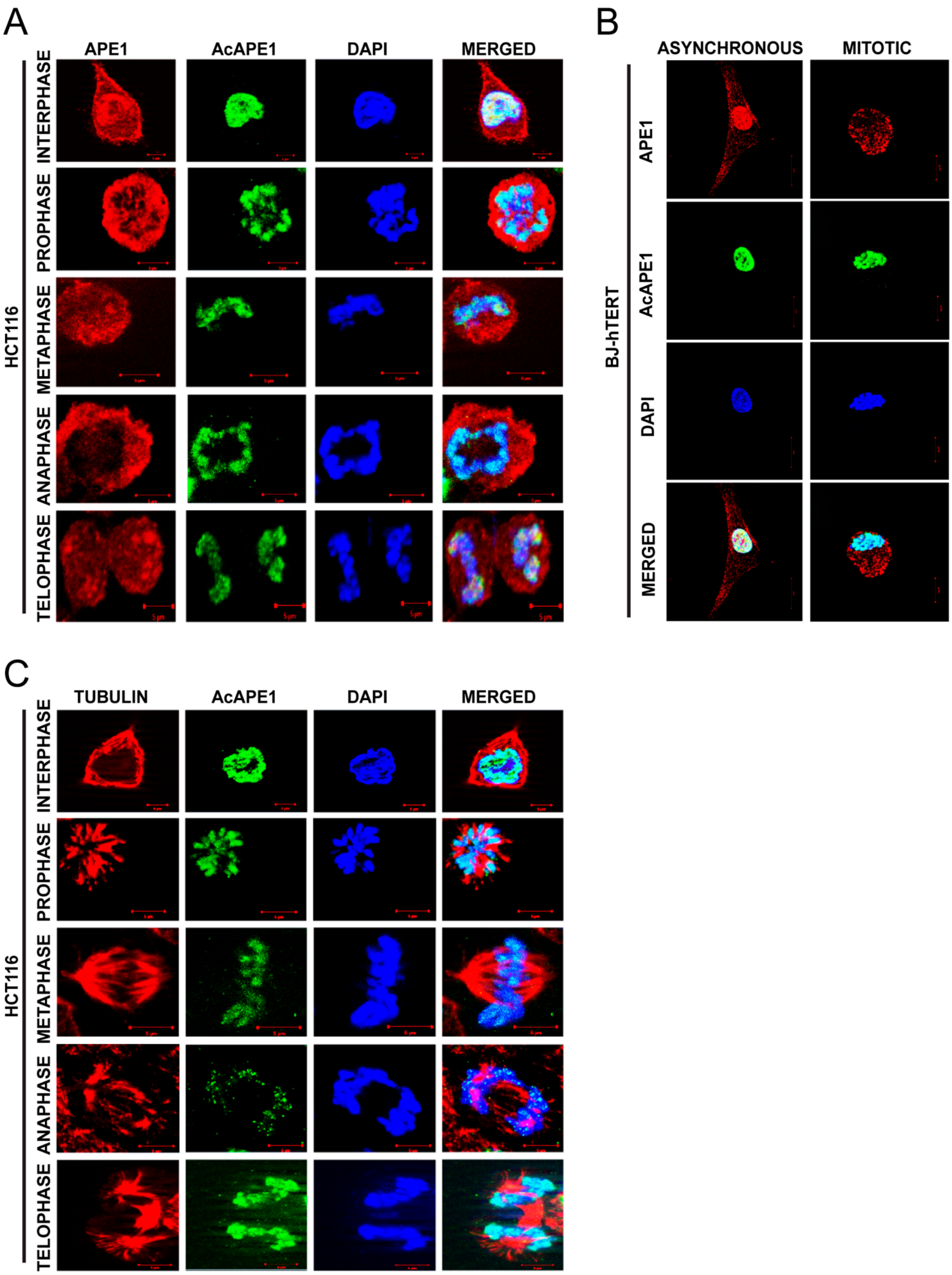

Figure S1. AcAPE1 is exclusively present on the chromatin throughout the cell cycle. (A) Confocal microscopy images of interphase and mitotic (different phases) HCT116 colon cancer cells immunostained with α-APE1 and α-AcAPE1 antibodies. Cells were counterstained with DAPI (Magnification 63X; scale bars 5 μm). **(B)** Confocal microscopy images of interphase and mitotic BJ-hTERT cells immunostained with α-APE1 and α-AcAPE1 antibodies. Cells were counterstained with DAPI (Magnification 63X; scale bars 5 μm). **(C)** Confocal microscopy images of interphase and mitotic (different phases) HCT116 colon cancer cells immunostained with α-tubulin and α-AcAPE1 antibodies. Cells were counterstained with DAPI (Magnification 63X; scale bars 5 μm).

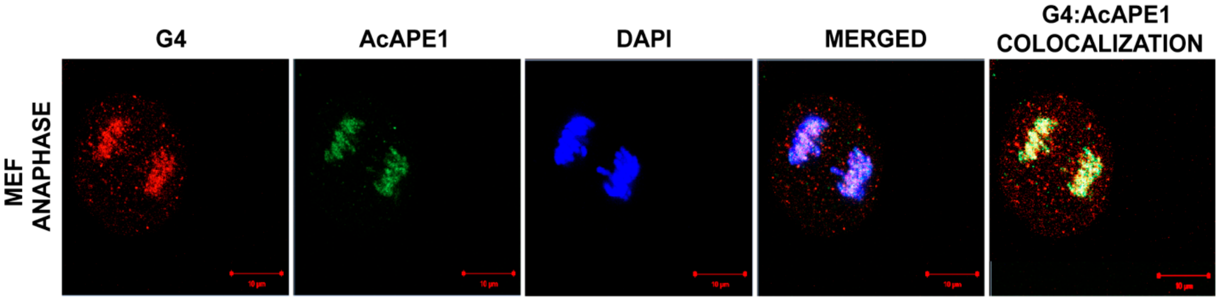

Figure S2. Colocalization of G4 and AcAPE1 staining in mitosis. Confocal microscopy image of anaphase arrested OGG1$^{+/+}$ MEF cell immunostained with α-1H6 and α-AcAPE1 antibodies. Cells were counterstained with DAPI (Magnification 63X; scale bars 10 μm).

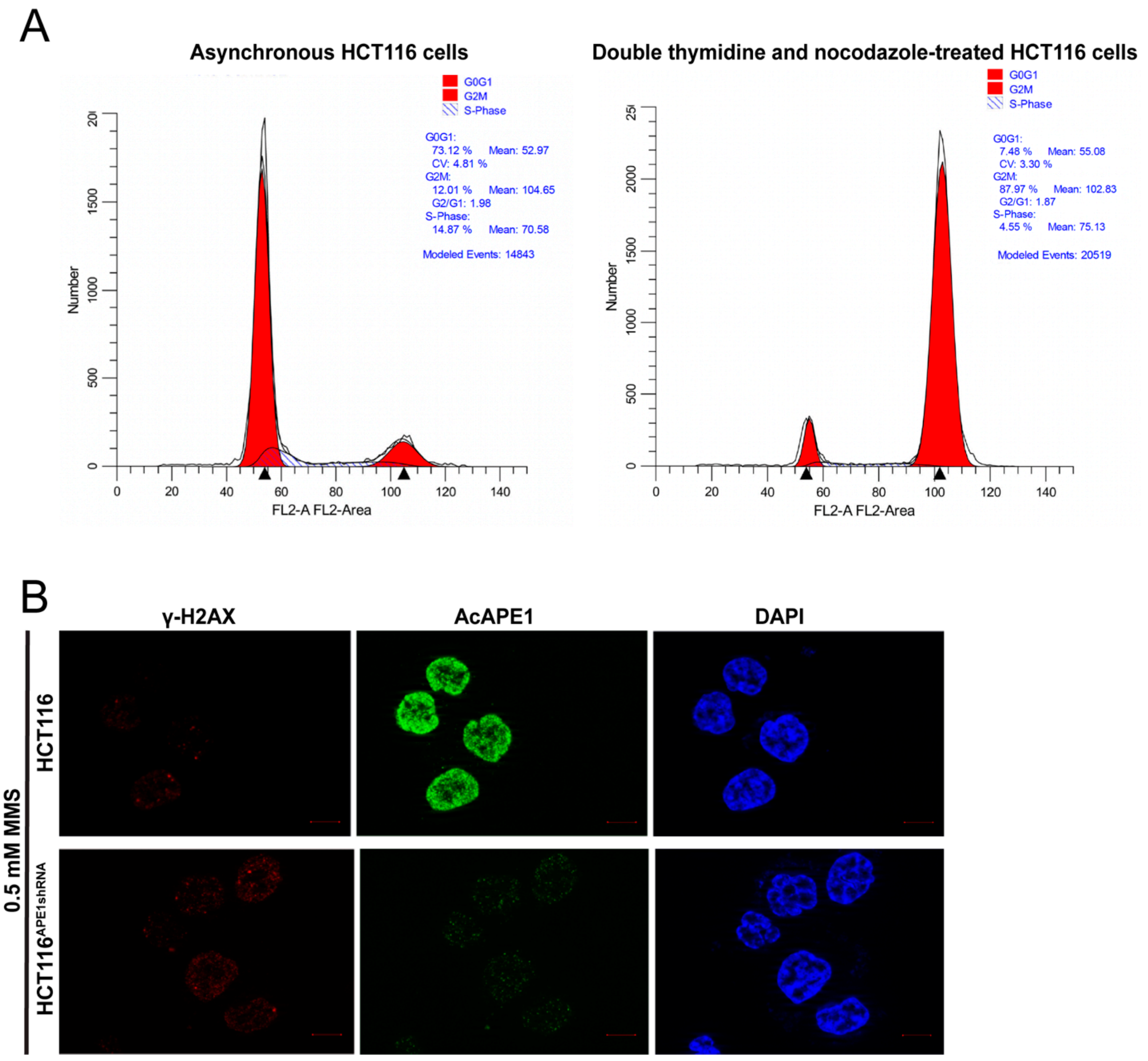

Figure S3. MMS treatment causes damages that are repaired primarily through BER.

Confocal microscopy images of HCT116 and HCT116[APE1shRNA] cells treated with 0.5 mM MMS for 15 minutes and immunostained with α-gamma H2AX and α-AcAPE1 antibodies. Cells were counterstained with DAPI (Magnification 63X; scale bars 5 μm).
